# In vitro performance of violet LED and argon plasma with oxygen on dentin bleaching

**DOI:** 10.1590/0103-644020256707

**Published:** 2025-12-08

**Authors:** Karen Milaré Seicento Aidar, Lara Maria Bueno Esteves, Paulo Henrique dos Santos, Henrico Badaoui Strazzi-Sahyon, Juliana Aparecida Delben, Vanessa do Nascimento, Bruno Mena Cadorin, Ticiane Cestari Fagundes, André Luiz Fraga Briso

**Affiliations:** 1 Department of Restorative Dentistry, São Paulo State University (UNESP), Araçatuba School of Dentistry. Araçatuba, SP, Brazil; 2 Department of Dental Materials and Prosthodontics, São Paulo State University (UNESP), Araçatuba School of Dentistry, Araçatuba, SP, Brazil; 3 Restorative Dentistry Area, University of Toronto, Faculty of Dentistry, Canadá; 4 School of Dentistry and Department of Prosthodontics and Periodontology, Bauru School of Dentistry, University of São Paulo (USP), Bauru, SP, Brazil; 5 Department of Dentistry, Western Paraná State University, Dental School (UNOESTE), Paraná, Brazil; 6 Department of Chemical, Fluminense Federal University (UFF), RJ, Brazil; 7 Department of Chemical, Laboratory 214, Federal University of Santa Catarina (UFSC), Center of Physical and Mathematical Sciences, SC, Brazil

**Keywords:** dentin, hydrogen peroxide, phototherapy, plasma gases, tooth bleaching

## Abstract

This in vitro study aimed to evaluate the effect of violet LED and argon plasma, associated or not with oxygen, on chromatic alteration, whitening index, temperature variation, wettability, surface energy, total free interaction energy, and adhesive strength in dentin tissue. Thus, 100 bovine dentin discs were allocated to 5 groups (n=20): C - control; APL - argon plasma; APL+O - argon plasma + 3% oxygen; LED - violet LED; HP - 35% hydrogen peroxide. In the first bleaching treatment for each technique, chromatic alteration (*Δ*E_00_), bleaching index (*Δ*W_ID_), and temperature variation were analyzed. Subsequently, the specimens were repolished, receiving the surface treatments again and designated for contact angle (^o^), surface energy (γs), and total free interaction energy (*Δ*G) analyses (n=5). In contrast, the remaining specimens were restored for bond strength analysis (n=15). From each specimen, four beams were obtained, two evaluated immediately and two after 10,000 thermal cycles. Bond strength data were subjected to 2-way repeated measures ANOVA, and the other analyses to 1-way ANOVA with Tukey's post hoc test (α=0.05). APL+O presented bleaching efficacy similar to the HP group, which, in turn, had the lowest bond strength values (p<0.05). LED was the only treatment that generated a temperature increase and showed a hydrophobic surface. APL+O presented lower contact angle values and higher *Δ*G values. APL and APL+O showed bond strength values that surpassed only those of the control group. It was concluded that plasma treatments were effective in dentin bleaching; however, none of the evaluated therapies managed to prevent the loss of bond strength.

## Resumo

Este estudo in vitro objetivou avaliar o efeito do LED violeta e do plasma de argônio, associado ou não ao oxigênio, na alteração cromática, índice de clareamento, variação de temperatura, molhabilidade, energia de superfície, energia total de interação livre e resistência adesiva no tecido dentinário. Assim, 100 discos de dentina bovina foram alocados em 5 grupos (n=20): C - controlo; APL - plasma de argônio; APL+O - plasma de argônio + 3% de oxigênio; LED - LED violeta; HP - peróxido de hidrogênio a 35%. No primeiro tratamento clareador de cada técnica, foram analisadas a alteração cromática (ΔE_00_), o índice de clareamento (ΔW_ID_) e a variação de temperatura. Posteriormente, os espécimes foram repolidos, recebendo novamente os tratamentos de superfície, sendo designados para análises de ângulo de contato (^o^), energia de superfície (γs) e energia de interação livre total (ΔG) (n=5), enquanto os espécimes restantes foram restaurados para análise de resistência de união (n=15). De cada espécime, foram obtidos quatro palitos, dois avaliados imediatamente e dois após 10.000 ciclos térmicos. Os dados de resistência de união foram submetidos a ANOVA de 2 vias de medidas repetidas e as outras análises a ANOVA de 1 via com teste post hoc de Tukey (α=0,05). APL+O apresentou eficácia clareadora semelhante ao grupo HP, que, por sua vez, apresentou os menores valores de resistência de união (p<0,05). O LED foi o único tratamento que gerou aumento de temperatura e apresentou superfície hidrofóbica. APL+O apresentou menores valores de ângulo de contato e maiores valores de ΔG. APL e APL+O apresentaram valores de resistência de união superados apenas pelo grupo Controle. Concluiu-se que os tratamentos com plasma foram eficazes no clareamento da dentina; no entanto, nenhuma das terapias avaliadas conseguiu evitar a perda de resistência de união.

**Figure:**
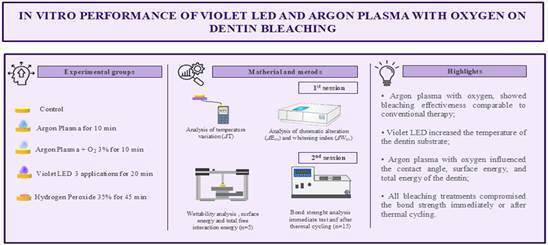

## Introduction

Restoring discolored substrates is a challenge, especially in anterior teeth, because it requires not only replicating appropriate contour, texture, and anatomical features but also correcting chromatic variations to ensure seamless integration with adjacent teeth, ensuring that they blend harmoniously with the surrounding dentition. For this reason, it is common to include whitening treatments in rehabilitative plans, increasing the probability of successful restorations.^1^

The traditional whitening protocol involves the application of hydrogen peroxide-based gels that release reactive oxygen species (ROS), which rapidly diffuse through dentinal tubules, oxidizing chromophore molecules and breaking them down into smaller and less pigmented compounds.^2^ ^,^ ^3^

However, the non-selective oxidation promoted by peroxides prevents restorations from being carried out immediately when the dentin remains darkened after cavity preparation, as the residual oxygen impedes monomer penetration and inhibits polymerization, requiring an interval of 7 to 21 days for the oxygen to be eliminated and for bond strength values to be restored.^3^ In addition, the direct application of peroxides to dentin is contraindicated due to its proximity to pulp tissue, leading to possible cytotoxic effects.^4^ Therefore, the ideal treatment in these cases would be a technique that modifies the color of the exposed dentin substrate, without the adverse effects of peroxides on the dentin-pulp complex and eliminates the prolonged waiting period before restorative procedures.^3^

In this context, violet light-emitting diodes (violet LEDs) have been studied for their photolytic action on pigment molecules. Emitting light at a wavelength of 405-410 nm, which corresponds to the absorption peak of chromophore molecules, violet LEDs can break down pigments even without the use of peroxides.^5^

The effects of violet LED have been examined in both *in vitro* and clinical settings, either alone or in combination with whitening gels. Studies have shown that its application leads to noticeable improvements in tooth color, particularly when used alongside low-concentration gels. (5-7) However, Kury et al.^7^ reported that violet LED has limited penetration in dental tissues, reaching a maximum depth of approximately 1 mm into the enamel. Due to this constraint, its whitening action remains confined to superficial layers, primarily affecting the areas closest to the light source.

Given these findings, researchers proposed applying LED directly to dentin tissue stained by different substances to simulate discoloration that may be present in dentin tissue exposed during cavity preparation. Their study confirmed that LED irradiation can significantly reduce dentin pigmentation, thereby improving esthetic outcomes in restorative procedures.^8^

Furthermore, Okada et al.^9^ and Kury et al.^10^ also evaluated an alternative approach to achieving whitening effects without the direct use of peroxides. In their studies, the authors investigated low-temperature plasma and observed its ability to whiten dental substrates. The whitening effect was attributed to the generation of hydroxyl radicals, which degrade chromophore molecules and can be used alone or in combination with whitening gels. Additionally, it was demonstrated that this therapy does not cause thermal damage or an inflammatory reaction in the pulp or oral soft tissues.^11^

The direct application of plasma to dentin tissue offers several potential benefits. Beyond its ability to induce chromatic alteration, it can also enhance the surface properties of dentin, improving its interaction with adhesive and restorative systems.^12^ Moreover, combining argon plasma with oxygen can boost the formation of reactive species, which serve as potent bactericidal agents in dentin tissue. This interaction may also create a synergistic effect in whitening therapy, further enhancing its efficacy by increasing chemical reactivity.^12^ ^,^ ^13^

Considering that these new protocols could provide significant aesthetic gains and enable the immediate execution of restorative procedures after whitening the dentin, conducting this study was deemed plausible. This *in vitro* study aimed to evaluate the effect of whitening techniques based on hydrogen peroxide, violet LED, and argon plasma with or without oxygen, on chromatic alteration (ΔE₀₀ and ΔW_ID_), temperature variation (ΔT), wettability (^o^), surface energy (γₛ), total free interaction energy (ΔG), and both immediate and post-thermal aging bond strength of resin-based materials applied to dentin tissue.

The null hypotheses tested were that there would be no difference in chromatic alteration or the whitening effect of dentin tissue subjected to different technique; There would be no difference in thermal alteration of dentin tissue subjected to the evaluated techniques; There would be no difference in wettability, surface energy, or total free interaction energy of dentin tissue subjected to the evaluated techniques; There would be no difference in the bond strength of dentin tissue subjected to the evaluated techniques; Thermal cycling would not affect the bond strength of dentin tissue subjected to the evaluated bleaching techniques. 

## Materials and methods

Prior to conducting the experimental analyses, this study received approval from the local Animal Use Ethics Committee (Process #682/2021). This *in vitro* study employed bovine tooth fragments, evaluating the following factors:

A) Whitening therapies, categorized into five levels: 1. No whitening treatment (C); 2. Argon plasma (APL); 3. Argon plasma combined with 3% oxygen (APL+O);4. Violet LED (LED) and 5. 35% hydrogen peroxide (HP). B) Bond strength evaluation, assessed at two distinct periods: 1. Immediately after treatment, and 2. Following 10,000 thermal cycles.

The response variables were chromatic alteration (ΔE_00_), whitening index (ΔW_ID_), temperature variation (ΔT), wettability, surface energy (γs), total free interaction energy (ΔG), and bond strength analyses.

### Specimen Acquisition

All materials employed in the study are detailed in Table 1.

**Table 1:** Material, trademark, manufacturer, composition, and batch number of the materials used.

Material	Trade Mark	Manufature	Composition	Batch
Black Tea	Chá Matte Leão	Coca-Cola Company (Curitiba, PR, Brazil)	Roasted mate leaves (*Ilex paraguariensis St. Hil.)*	23117
Whitening Gel based on 35% Hydrogen Peroxide	Whiteness HP	FGM (Joinville, SC, Brazil)	35% hydrogen peroxide, thickeners, dye mixtures, glycol, inorganic filler, and deionized water.	311022
35% Phosphoric Acid	3M™ Scotchbond™	3M/ESPE (St. Paul, MO, USA)	35% phosphoric acid, thickening agent composed of pyrogenic silica and water-soluble surfactant.	1021
Adhesive System	Adper Single Bond 2	3M/ESPE (St. Paul, MO, USA)	BisGMA, HEMA, 1,3-glycerol dimethacrylate, diurethane dimethacrylate, water, ethanol, photoinitiators, silanized silica 5 nm, polyacrylic and itaconic acid copolymer.	2307900366
Composite Resin	Filtek Z250 XT	3M/ESPE (St. Paul, MO, USA)	BisGMA, TEGDMA, Bisphenol A polyethylene glycol diether dimethacrylate, UDMA, treated silanized ceramic, and silane-treated silica.	2320200807

Abbreviations: Bis-GMA, bisphenol-A glycidyl methacrylate; HEMA, 2-hydroxyethylmethacrylate; TEGDMA, triethylene glycol dimethacrylate; UDMA, urethane dimethacrylate.

Initially, bovine incisors without stains, excessive wear, crown alterations, or enamel cracks were selected from animals aged 30 to 40 months. The teeth were cleaned with periodontal curettes and submitted to a prophylaxis with a silicone rubber cup, pumice stone, and water. To avoid bacterial proliferation, the teeth were stored in a saline solution with 0.1% thymol and kept in a refrigerator at 4°C until the start of the experimental phase.^8^

After this, the roots were separated from the crowns at the cementoenamel junction. The crowns were secured in a device connected to the platform of a bench drill (model FGC-16, Ferrari, São Paulo, SP, Brazil) and perforated using a diamond trephine bur (Dinser Diamond Tools Ltda, Sacomã, SP, Brazil) to obtain 5.7 mm diameter enamel/dentin disks from the middle third of the buccal surface.

After obtaining the discs, the specimen surface was refined by manual rotational polishing with #400 and #600 grit aluminum oxide sandpaper (T469-SF-Noton, Saint-Gobain Abrasives Ltda, Jundiaí, SP, Brazil) until a thickness of 4.0 mm was achieved (comprising 1.2 mm of enamel and 2.8 mm of dentin ± 0.2 mm), measured with a digital caliper (model 500-144B, Mitutoyo Sul América Ltda, São Paulo, SP, Brazil).

### Specimen Selection and Experimental Groups

During the pigmentation process, each specimen was stored in polypropylene tubes (Eppendorf, Hamburg, Germany) containing one mL of black tea infusion at room temperature and kept at 37^o^C in an incubator for 24 hours. The infusion was prepared with 1.6 g of black tea (Matte Leão Tea, Curitiba, PR, Brazil) per 200 mL of distilled water. After this period, to remove excess pigments that had not been incorporated into the dental structure, the specimens were washed and subsequently immersed for 48 hours in distilled water and kept in an oven at 37^o^C.

After pigmentation, the specimens were affixed to an acrylic base with a 2 mm stopper, and the enamel layer was removed using a #400 and #600 sandpaper in a polishing machine (Aropol E, Arotec, Cotia, SP, Brazil), resulting in dentin discs with a thickness of 2 mm.^8^ At the end of this stage, the specimens underwent a 10-minute immersion in an ultrasonic bath with distilled water. The complete removal of the enamel layer was verified using a stereoscopic loupe (Carl Zeiss, Oberkochen, Germany) at magnifications of ×6 and ×60. The dentin discs were placed in a two mm-thick black silicone matrix (5.7 mm in diameter), which featured a reference mark to standardize specimen positioning across all readings.

An initial reading (T0) was performed using a Visible Ultraviolet Reflection Spectrophotometer (Model UV-2450, Shimadzu, Kyoto, Japan), which employs the CIELab system, allowing for color perception in a three-dimensional model. The L* coordinate represents luminosity, ranging from 0 (black) to 100 (pure white). The a* coordinate indicates the proportion of red (positive values) and green (negative values), while the b* coordinate represents the concentration of yellow (positive values) and blue (negative values).

After the readings, 100 specimens were selected based on their L values*, choosing those closest to the overall mean (61.21 ± 5.93). The selected specimens were divided into five experimental groups (n=20), as presented below.

**Figure 1:**
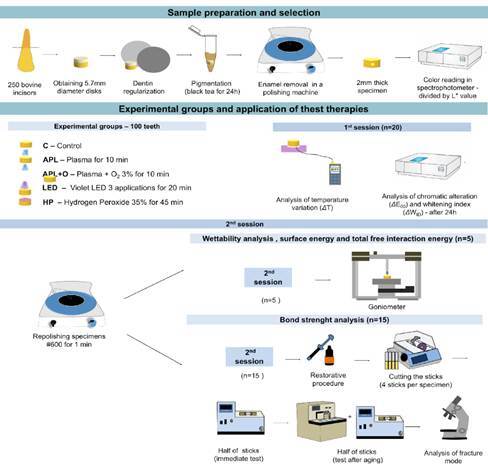
Flowchart of the experimental design.

The first treatment application aimed to analyze chromatic alteration (ΔE_00_), whitening index (ΔW_ID_), and temperature variation (ΔT) (n=20). After this stage, a polishing procedure was performed to remove any residual treatment effects, allowing the specimens to be reused in subsequent analyses. In the second phase, 25 specimens (n=5 per group) were allocated to evaluate contact angle, surface energy, and total free interaction energy (ΔG). The remaining 75 specimens (n=15 per group) were designated for bond strength analysis (Figure 1).

### Application of Test Therapies

The division of groups and whitening treatments were performed according to the protocols established as detailed below:

C (Control): This group underwent the same pigmentation and abrasion protocols as the experimental groups described previously; however, no whitening intervention was performed.

APL (Argon Cold Plasma): Argon cold plasma was applied using a Plasma Pen device (WIER Plasma and Ozone Technology, Florianópolis, Brazil), specifically designed for dental applications in research settings. This portable unit was connected to a high-frequency power generator that produces plasma under atmospheric conditions. An acrylic support was attached to the portable unit to standardize the distance between the dentin surface and the plasma source. The device ionizes the argon gas, generating plasma at the electrode tip, which is then directed onto the specimen. The argon gas flow rate was maintained at 1.5 L/min, controlled by a flow regulator.^12^ The distance between the plasma-emitting tip and the specimen was set at 1 cm. Plasma was applied for 10 minutes^10^ with continuous movement of each specimen using plastic tweezers to ensure uniform exposure of the entire surface.

APL+O (Argon Cold Plasma with Oxygen): This group underwent the same treatment protocol as the APL group; however, 3% O₂ gas was added at a flow rate of 45 mL/min. As in the APL group, the argon flow rate was maintained at 1.5 L/min^12^ with a fixed plasma-specimen distance of 1 cm, for a total application time of 10 minutes^10^.

LED (Violet LED Light): The specimens were treated with violet LED light using the Bright Maxx Whitening Photocatalyst (MMOptics Ltda., São Carlos, SP, Brazil). This device emits light from four LED diodes, with a wavelength range of 405-410 nm and a maximum total optical power of 1.5 W. The treatment protocol consisted of three 20-minute applications, with 10-minute intervals between each session. The device features a delivery system with an anatomically designed, polished acrylic tip that homogenizes and collimates the emitted beams, ensuring uniform delivery over a defined area of 10.7 cm², resulting in an irradiance of 140.2 mW/cm². The device was positioned 1 cm away from the specimens throughout the application period^8^.

HP (Hydrogen Peroxide): The whitening agent Whiteness HP (FGM Dental Products, Joinville, Santa Catarina, Brazil), containing 35% hydrogen peroxide, was prepared according to the manufacturer's instructions (three drops of hydrogen peroxide to one drop of thickener). The mixture was transferred to a micropipette for viscous liquids (Microman® E, Gilson, West Beltline Hwy, Middleton, USA), and 0.05 mL of the resulting gel was evenly applied to the dentin specimen. The gel remained in contact with the specimen for 45 minutes, with replacements every 15 minutes. At each change, the gel was removed using sterile gauze and absorbent papers, then rinsed with distilled water.

In the study design, the HP group served a dual purpose: it functioned as the positive control for evaluating the whitening effect and as the negative control for bond strength testing. Conversely, the C group served as the positive control for bond strength assessment and as the negative control for whitening evaluation. It is important to highlight that throughout the experiment, the specimens were maintained in a humidifying chamber in an oven at 37°C.

### Analysis of Chromatic Alteration (∆E_00_)

After the application of the treatment, all 20 specimens from each group were stored in a humidified chamber at 37°C and 100% humidity for 24 hours. After this incubation period, spectrophotometric analysis (T1) was performed, employing the same methodology previously described and compared to baseline values (T0). Chromatic alteration values were then calculated using the formula^14^:

$$∆E_{00}=\;{\left[{\left(\frac{∆L^{'}}{K_{L\;}S_{L}}\right)}^{2}+\;{\left(\frac{∆C^{'}}{K_{C\;}S_{C}}\right)}^{2}+{\left(\frac{∆H^{'}}{K_{H\;}S_{H}}\right)}^{2}+R_{T\;}\left(\frac{∆C^{'}}{K_{C\;}S_{C}}\right)\left(\frac{∆H^{'}}{K_{H\;}S_{H}}\right)\right]}^{\frac{1}{2}}$$

This formula accounts for variations in luminosity (*∆*L*), saturation (*∆*C*), and hue (*∆*H*) by incorporating luminosity compensation coefficients (SL), chroma (SC), and hue (SH). It also includes ΔR, an interaction term between saturation and hue differences, providing a more accurate alignment with human visual perception^14^.

### Analysis of Whitening Index (∆W_ID_)

As an additional analysis, the whiteness index (W_ID_) was also investigated. This index is calculated using a linear formula based on the values of the three CIELab chromatic coordinates. It was calculated by comparing the values from the first application (T1) to the baseline (T0) using the formula below^15^:

WID = (0,511 x L*) - (2,324 x a*) - (1,100 x b*)

Positive W_ID_ values indicate a greater whitening effect in the specimens, while lower or negative values indicate a reduced whitening effect. The W_ID_ values were also interpreted based on the criteria established, which determined a value of 0.72 as the perceptibility threshold and 2.6 as the acceptability threshold^16^.

### Analysis of Temperature Variation (ΔT)

During the first application of the treatments, performed to evaluate chromatic alteration and whitening index, the specimens were coupled to a silicone matrix to evaluate temperature variation. A silicone matrix with four perforations, each measuring 5.7 mm in diameter and 2.5 mm in depth, was fabricated for temperature variation analysis. The specimens were positioned within these perforations, with the smaller openings facing the inner surface. Type K thermocouple terminals (Risepro, Kowloon, Hong Kong, China) with a precision of ±0.05°C were inserted through these openings to ensure accurate temperature monitoring. To optimize thermal conductivity, the interface between the inner surface of the dentin disc and the thermocouple terminal was coated with thermal paste (Implastec, Votorantim Ind. Brasileira, São Paulo, SP, Brazil). Temperature variation was continuously monitored in real time throughout the first treatment application. The recorded data included the difference between the maximum temperature reached and the initial baseline temperature^8^.

### Wettability analysis, surface energy, and total free interaction energy

Twenty-five specimens (n = 5) initially utilized for temperature variation and color alteration analyses were subsequently used to evaluate wettability, surface energy, and total free interaction energy. To prepare for these analyses, the dentin surfaces previously exposed to the whitening treatments were polished using #600-grit aluminum oxide sandpaper, attached to a polishing machine, for 1 minute under water cooling. Afterward, the specimens were cleaned in an ultrasonic bath for 10 minutes. The refreshed surfaces were then re-exposed to the same surface treatment procedures before undergoing evaluations.^12^

The free surface energy (γs) of the dentin, along with its non-polar (γLW: Lifshitz-van der Waals) and polar (γAB: acid/base) components, was assessed by measuring the contact angle immediately after the second application of the whitening treatments. Measurements were performed using an automatic goniometer (DSA 100S, Krüss, Hamburg, Germany) with three liquids of established surface energy parameters: water (polar), methylene iodide (non-polar), and ethylene glycol (polar with acid/base characteristics).^12^

After surface treatment, 0.3 μL of each liquid was dispensed onto the surface of each specimen in three predetermined areas specific to each liquid, using a 500 μL glass syringe equipped with a 0.5 mm gauge needle. The contact angle was measured by capturing the droplet's image using Drop Shape Analysis DSA4 Software (version 2.0-01; Krüss, Hamburg, Germany) and determining the angle with the tangent method. Each droplet was measured five times at 5-second intervals under controlled conditions of 20°C and a relative humidity of 44% ± 6, to calculate an average value.^12^

The evaluation of surface free energy (mN/m) was based on the van Oss, Chaudhury, and Good model, which considers Lifshitz-van der Waals forces (γLW, non-polar component), Lewis acid-base interactions (γAB, polar component), and acid (γ+, receptor) and base (γ−, donor) components.^17^

This approach enabled the precise determination of the free energy values of the substrates, using the following equation^18^:

$$\left(1+cos\;\theta \right)\gamma_{s}=-2\left({\sqrt{\gamma_{s}}}^{LW}-{\sqrt{\gamma_{L}}}^{LW}\right)+({\sqrt{\gamma_{s}}}^{+}{\gamma L}^{-}+{\surd \gamma_{s}}^{-}{\gamma L}^{+})$$

The total free interaction energy (ΔG) between water and dentin was computed to determine the hydrophobicity or hydrophilicity of the surface, using the equation^18^.

$$\Delta G\;=\;-2\;{\left({\sqrt{\gamma_{s}}}^{LW}-{{\sqrt{\gamma }}_{w}}^{LW}\right)}^{2}-4\left({{\sqrt{\gamma }}_{s}}^{+}{\gamma_{s}}^{-}+{{\sqrt{\gamma }}_{w}}^{+}{\gamma_{w}}^{-}-{{\sqrt{\gamma }}_{s}}^{+}{\gamma_{w}}^{-}-{{\sqrt{\gamma }}_{s}}^{-}{\gamma_{w}}^{+}\right)$$

When ΔG is greater than 0, it indicates that the surface is hydrophilic, and when it is less than 0, the surface is characterized as hydrophobic.

### Microtensile Bond Strength Analysis

The remaining 75 specimens (n=15) underwent the same abrasion procedure as previously described before restoration. Each specimen was individually subjected to the bleaching protocol, and immediately after completing the treatment, the adhesive procedures were carried out. Thus, the dentin surface exposed to the treatments was etched with 35% phosphoric acid (3M ESPE, St. Paul, MN, USA) for 15 seconds, then rinsed with water for 15 seconds and dried while maintaining dentin moisture.

After acid etching, the Single Bond 2 adhesive system (3M ESPE, St. Paul, MN, USA) was applied in two successive layers to the etched area, following the manufacturer's instructions. The adhesive was gently air-dried for 5 seconds and light-cured for 10 seconds using the VALO LED Cordless unit (Ultradent, South Jordan, UT, USA) set to standard mode (1000 mW/cm^2^). The specimens were then placed in a silicone mold matching their diameter, with a height of 4 mm. Filtek Z250 XT composite resin (3M ESPE, St. Paul, MN, USA) was inserted incrementally and light-cured for 20 seconds.

The restored specimens (n=15) were stored in a humidifying chamber inside an oven at 37ºC. After 48 hours, they were sectioned using a metallographic cutting machine (Isomet 1000, Buehler, Lake Bluff, IL, USA) along the buccal-lingual and mesio-distal directions at 250 rpm under water cooling to prevent overheating. This process yielded at least four rectangular beams (each≅1×1 mm and 6mm in height) from the core of the blocks, measured with a digital caliper (Mitutoyo, São Paulo, SP, Brazil).

Immediately after sectioning, two beams were subjected to a microtensile bond strength test, while the remaining two underwent thermal cycling to simulate oral temperature variations. The remaining beams were subjected to 10,000 thermal cycles, alternating 30-second immersions at 5°C and 55°C, with a 2-second transfer period.^19^ ^,^ ^20^

**Figure 3:**
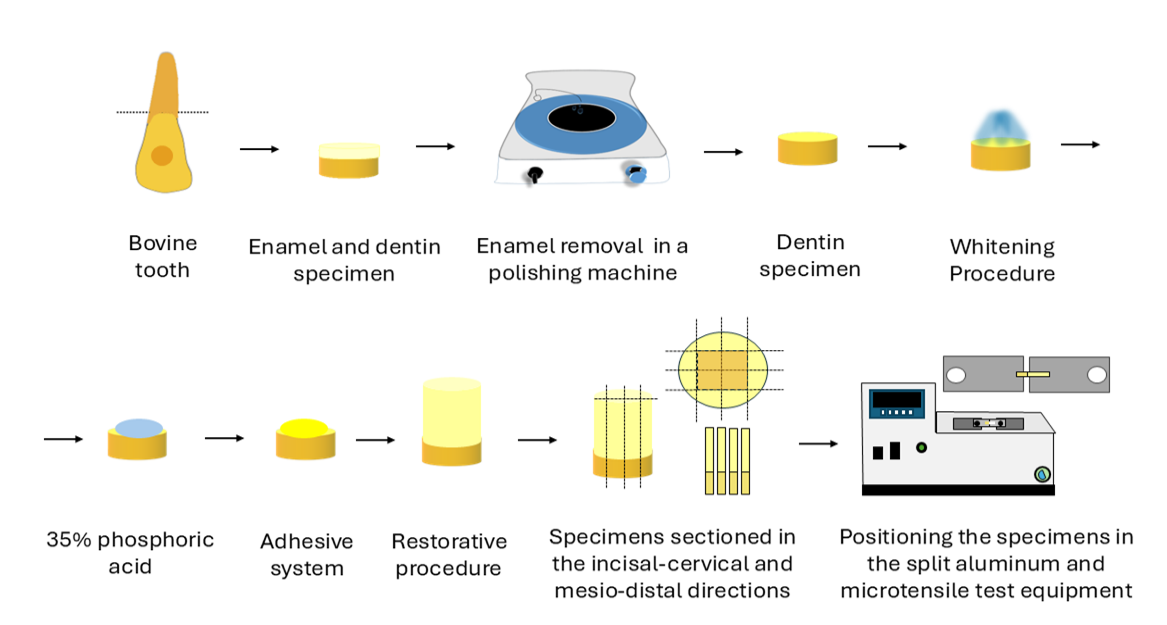
Detailed flowchart of the Microtensile Bond Strength Analysis.

The specimens were positioned in a split aluminum microtensile test equipment (Odeme Dental Research, Luzerna, SC, Brazil), equipped with a central groove explicitly designed for accommodating the beams, which ensured precise retention of the specimen by directing the load parallel to the adhesive interface. To do this, the ends of the beams were fixed to the device with cyanoacrylate adhesive (Super Bonder Power Flex Gel, Henkel Ltda., Brazil), ensuring precise positioning of the adhesive interface in the center of the metal stub without any additional securing material. The devices containing the beams were mounted in an Odeme Microtensile OM 100 testing machine (Odeme Dental Research, Luzerna, SC, Brazil), operating at 0.7 mm/min to assess the force required to fracture the specimen (N).^18^ Bond strength measurements (MPa) were calculated using the equation: BS = F/A, where BS is bond strength (MPa), F is the maximum force (N), and A is the bond interface area (mm^2^). Figure 3 below illustrates the detailed flowchart of the Microtensile Bond Strength Analysis.

After rupture, the fragments were examined under a stereomicroscope (Carl Zeiss, Oberkochen, Germany) at ×60 magnification to classify the fracture mode as adhesive failure, cohesive failure in dentin, cohesive failure in the restorative material, cohesive failure in the adhesive layer, or mixed failure.^19^ To analyze the bond strength values, the dentin specimen was considered the experimental unit at each analysis time. When two beams were obtained from a single specimen, the average of the bond strength values was calculated, and this value was used in the statistical analysis. In cases where no beam could be obtained from a specimen, a value of zero was assigned to represent premature failure. Conversely, when only one beam could be obtained from a specimen, its value was halved to maintain the consistency of the weighting between all the specimens analyzed.^20^

### Statistical Analysis

The descriptive and exploratory data analysis confirmed that the assumptions of normality and homogeneity were met. A one-way ANOVA was performed to analyze temperature, chromatic alteration (ΔE_00_), whitening index (ΔW_ID_), wettability, surface energy (γs), and total free interaction energy (ΔG). A two-way repeated measures ANOVA was used to assess bond strength. All analyses were subjected to Tukey's post-hoc test at a 5% significance level, using SigmaPlot 12.0 statistical software.

## Results

### **Chromatic Alteration and Whitening Index *(*Δ*E***_*00*_ ***e* Δ*W***_*ID*_ ***)***

Table 2 presents the analysis of chromatic alteration (ΔE_00_) and whitening index (ΔW_ID_) following different therapies. For ΔE_00_, the highest values were observed in APL, APL+O, and HP groups, with statistically significant differences compared to the LED and C groups, which exhibited the lowest values (p<0.05). The LED group showed higher ΔE_00_ values than the C group (p<0.001).

Regarding ΔW_ID_, the most pronounced alteration was observed in the HP group; however, no statistically significant difference was found compared to the APL+O (p=0.146). HP and APL+O showed a significant difference compared to the other experimental groups (p<0.001). APL showed higher values and a statistically significant difference compared to LED, with both differing significantly from the C group (p < 0.001).

**Table 2:** Mean (standard deviation) of *Δ*E_00_ and *Δ*W_ID_ values obtained after sample pigmentation (T1) compared with data observed after bleaching (T2).

Groups	Optical Properties	
	***Δ*E**_00_	***Δ*W**_ID_
C	1.56 (0.56) C	0.92 (0.74) D
APL	11.21 (3.89) A	6.88 (2.01) BC
APL+O	11.02 (4.06) A	8.91(1.71) AB
LED	5.60 (2.48) B	4.22 (1.32) CD
HP	10.08 (2.32) A	10.11 (2.03) A

Distinct uppercase letters in the column indicate statistically significant differences for each investigated optical property (p<0.05).

**Figure 2:**
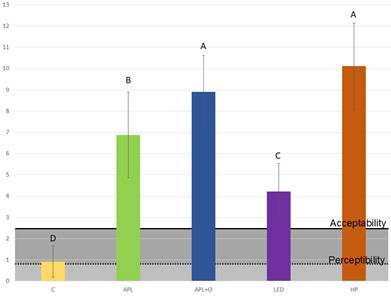
Presentation of the ΔW_ID_ values in all groups according to the acceptability (2.62) and perceptibility (0.72) limits obtained after the bleaching session.

Figure 2 illustrates the ΔW_ID_ values for all groups following bleaching, categorized by acceptability and perceptibility thresholds. All test therapies exceeded the acceptability threshold, with the highest values observed in the HP group. Conversely, the C group only surpassed the perceptibility threshold.

### Temperature Variation (ΔT)

The temperature variation results are presented in Table 3, showing that the LED group exhibited the highest thermal change, with a statistically significant difference compared to the other experimental groups (p<0.05). However, no significant differences were observed among the C, APL, APL+O, and HP groups (p>0.05).

**Table 3:** Mean (standard deviation) of *Δ*T (ºC) values obtained during the bleaching treatment.

Groups	***Δ*T (ºC)**
C	0.15 (0.11) B
APL	-0.32 (1.02) B
APL+O	0.18 (0.74) B
LED	4.34 (0.86) A
HP	0.04 (0.06) B

Different uppercase letters in the column indicate statistically significant differences (p<0.05).

### Wettability (º), Surface Energy (γs), and Total Free Interaction Energy (ΔG)

Table 4 presents data on contact angle, surface energy, and total free interaction energy following bleaching treatments. The LED group exhibited the highest contact angle values, with a statistically significant difference compared to all other experimental groups (p<0.05). The C group showed higher contact angle values than the APL, APL+O, and HP groups (p<0.05). While the APL and APL+O groups differed significantly from each other (p=0.004), neither showed a statistically significant difference compared to the HP group (p>0.05).

**Table 4:** Mean (standard deviation) values of the contact angle (º), surface energy - γs (mN/m), and total free interaction energy - *Δ*G (mJ/m^2^) of the dentin surface according to different bleaching treatment protocols.

Groups	Parameters		
	Contact Angle	Surface Energy	***Δ*G**
C	69.74 (4.77) B	14.78 (8.98) A	6.03 (4.32) D
APL	48.70 (7.27) C	18.04 (15.13) A	23.93 (14.88) C
APL+O	28.82 (8.58) D	24.90 (10.94) A	64.89 (3.16) A
LED	86.50 (6.57) A	22.26 (4.91) A	-24.37 (6.92) E
HP	42.88 (9.54) CD	15.30 (13.00) A	42.78 (9.60) B

Distinct uppercase letters in the columns indicate statistically significant differences for each evaluated parameter (p<0.05).

Regarding surface energy, all experimental groups presented statistically similar results (p>0.05). However, in the analysis of total free interaction energy (ΔG), statistically significant differences were observed among all experimental groups (p<0.05). The APL+O group exhibited the highest ΔG values, followed in descending order by HP, APL, C, and LED. Notably, the LED group had negative ΔG values, indicating a hydrophobic surface.

### Bond Strength Analysis

Table 5 presents the bond strength data obtained before and after thermocycling. In the immediate analysis, all treatments were found to negatively influence bond strength values, with statistically significant differences compared to the C group (p<0.05). Although the APL and APL+O groups did not differ statistically from each other (p=0.819), both exhibited higher bond strength values than the LED and HP groups (p<0.05). The HP group, which demonstrated the lowest bond strength values, also showed a statistically significant difference compared to the LED group (p<0.001).

**Table 5:** Mean (standard deviation) of bond strength values (MPa) in relation to the bleaching treatments and aging periods.

Groups	Periods	
	Immediate	After Thermocycling
C	23.41 (4.27) Aa	18.57 (3.62) Ab
APL	18.10 (1.51) Ba	10.50 (2.36) Bb
APL+O	19.08 (5.57) Ba	11.99 (3.49) Bb
LED	13.71 (4.51) Ca	8.67 (2.84) BCb
HP	4.73 (3.02) Da	3.86 (2.44) Ca

Different uppercase letters in the columns and lowercase letters in the rows indicate statistically significant differences (p<0.05).

A similar trend was observed after thermocycling; however, the LED group showed no statistically significant differences compared to the APL, APL+O, and HP groups (p>0.05). Additionally, except for the HP group (p=0.750), all other experimental groups exhibited a significant reduction in bond strength values after thermocycling (p<0.05).

The analysis of failure patterns revealed that in most experimental groups, adhesive failure was predominant, except in the HP group, which showed a higher incidence of premature failures both before and after thermal aging (Table 6). The control group exhibited the lowest number of premature failures, with cohesive failures observed in the restoration immediately and mixed failures after thermocycling. No experimental group exhibited cohesive failures in dentin tissue or the adhesive layer during any of the evaluated periods.

**Table 6:** Distribution of failure types according to the experimental group and aging periods.

Groups	Fracture mode							
	Period Evaluated	Premature Failure	Adhesive Failure	Cohesive In Dentine	Cohesive in the restoration	Cohesive in the Adhesive layer	Mixed Failure	Total
C	Immediate	2	19	0	9	0	0	60
	After Aging	1	25	0	1	0	3	
APL	Immediate	8	17	0	4	0	1	60
	After Aging	10	18	0	1	0	1	
APL+O	Immediate	3	17	0	6	0	4	60
	After Aging	6	17	0	3	0	4	
LED	Immediate	10	16	0	2	0	2	60
	After Aging	11	14	0	4	0	1	
HP	Immediate	19	10	0	0	0	1	60
	After Aging	20	9	0	0	0	1	

## Discussion

Traditional dental bleaching protocols have been shown to negatively impact the adhesion of resin materials, limiting immediate restorations after bleaching. Consequently, alternative treatments that avoid or eliminate peroxide-bleached tooth structures have been proposed.^7^ ^,^ ^8^ The efficacy of these treatments is increasingly evaluated using various methods, with the CIELab model gaining popularity for assessing chromatic alterations along individual color axes (a*, b*, and L*) or through combined formulas such as ΔE_00_ and ΔW_ID_.

Recent advancements include perceptibility and acceptability thresholds, offering a clinically relevant perspective on color changes. The perceptibility threshold represents the most minor clinically noticeable color variation, while the acceptability threshold defines the maximum degree of alteration deemed acceptable. Pérez et al.^15^ established 0.72 as the perceptibility threshold and 2.62 as the acceptability threshold for ΔW_ID_. The results showed that the experimental groups presented chromatic changes consistent with a noticeable whitening effect, exhibiting ΔW_ID_ values above the limit of clinical acceptability and the highest values observed in the HP group. Thus, there was a significant difference between the treatments, which led to the rejection of the study's first null hypothesis. However, a variation was observed in the W_ID_ of group C, with values slightly above the perceptibility threshold, but below the acceptability. This difference may be associated with the storage and handling of the specimens during the experimental period, including the dehydration of the surface and the partial degradation of the organic dental component, which can influence the color variation.^21^

Additionally, treatments involving argon plasma, with or without oxygen, produced a whitening effect; however, only APL+O achieved a whitening effect comparable to HP. The immediate dehydration observed in the specimens after the bleaching therapies required a 24-hour delay in the color readings to allow for their rehydration. The dehydration that occurred, in addition to being a methodological limitation of *in vitro* studies, may limit the clinical application of the study, since it complicates the selection of the color of restorative materials on a recently treated surface. In addition, it can compromise the integrity of the dentin-pulp complex, potentially leading to post-operative sensitivity.

Regarding the findings in the APL+O group, Choi et al.^22^ reported that combining argon with oxygen enhances the formation of reactive species, intensifying the bleaching effect (increase in *L* and reduction in *b* values). Their study used 50% oxygen, in contrast to the 3% applied in the present study, suggesting that higher oxygen concentrations could further enhance the bleaching effect. It is noteworthy that the oxygen concentration used in this study was based on previous research indicating that argon plasma combined with low oxygen concentrations significantly improves dentin surface reactivity, without altering its morphology, roughness, or chemical composition, which favors hydrophilicity and potentially improves restoration performance.^12^

However, Okada et al.^9^ and Kury et al.^10^ observed that plasma application alone resulted in a clinically perceptible whitening effect, although numerically lower than the values reported in the present study. These discrepancies may be attributed to differences in specimen types, as the previous studies evaluated entire crowns, whereas the present study used 2-mm-thick dentin discs. The permeability and water retention of this tissue may have contributed to a greater formation of hydroxyl radicals, generated by the interaction of plasma-generated electrons with water molecules present in the specimens.^11^

In the evaluation of chromatic alterations, the present study demonstrated that violet LED light induces significant changes in the substrate’s color, with ΔW_ID_ values exceeding both perceptibility and acceptability thresholds. These findings suggest that this technology is capable of promoting a clinically relevant whitening effect from the first bleaching session. The results are consistent with those reported in previous studies by Gallinari et al. (2020)^6^, Clemente et al. (2022)^8^, and Kury et al. (2020)^10^.

It is postulated that violet LED affects yellowish pigments by destabilizing them, disrupting carbon double bonds, and leading to a whitening effect. However, it was reported that violet LED performance may be reduced on blood-pigmented substrates due to the proximity of iron atoms, which decrease light photon transmission.^8^ This finding suggests a limitation of violet LED for cases of pigmentation caused by pulp hemorrhages. In contrast, plasma has been shown to effectively whiten blood-pigmented substrates through the release of ferrous ions, which contribute to hydroxyl radical formation.^23^

In the thermal variation analysis, only the LED group exhibited inconsistent temperature regulation, leading to the rejection of the second null hypothesis. Prolonged violet LED exposure resulted in a significant temperature increase (ΔT = 4.3°C), which could potentially cause moderate and reversible histopathological damage.^24^ This thermal effect observed with prolonged violet LED exposure is consistent with the findings of an *in vitro* study^8^ and supports the reported sensitivity described in clinical investigation.^6^ In contrast, argon plasma, regardless of oxygen association, did not induce a significant temperature increase, as plasma ionization heats electrons while maintaining ions and neutral particles at ambient temperature.^8^

After analyzing the surface data, it was evident that all treatments influenced contact angle, surface energy, and total free interaction energy, leading to the rejection of the third null hypothesis. However, immediate goniometric analysis after therapy application was not feasible due to the equipment setup time, which typically required 15 to 20 minutes.

Plasma treatment generates radicals and energy capable of modifying surface energy and hydrophilicity by removing hydrocarbons and introducing hydroxyl groups.^12^ In this context, the APL+O group demonstrated superior results, reducing the water contact angle and increasing total free interaction energy, indicating a highly hydrophilic surface. These findings align with previous studies^12^ ^,^ ^13^, which suggest that oxygen addition to plasma enhances its ability to modify dentin surface properties. This enhancement increases the electrostatic component of Lewis bases, facilitating interactions with electron-capturing components and promoting hydrogen bonding between the adhesive system and the dentin surface.^12^ ^,^ ^13^

The prolonged exposure and temperature increase observed in the violet LED group may have negatively affected the surface properties, reducing wettability, as indicated by the higher contact angle and negative values for free surface interaction energy. This suggests a more hydrophobic dentin surface, which could hinder water interaction and reduce adhesion with the hydrophilic adhesive system.^19^ Regarding the HP group, there are no reports in the literature on this specific type of surface analysis in dentin. However, it is well established that H₂O₂ affects dentin chemical stability, leading to carbonate and protein proteolysis, as well as collagen fiber degradation due to its oxidizing properties.^25^

In terms of bond strength, it was observed that the whitening therapies impacted the adhesive strength in dentin tissue, leading to the rejection of the study's fourth null hypothesis. The C group exhibited the highest values, consistent with the literature, which aligns with the traditional restorative technique used.^26^ Conversely, the HP group showed reduced bond strength, reflecting the high incidence of premature failures, as previously reported in studies that performed immediate restorations after bleaching.^4^ ^,^ ^26^ It was demonstrated that residual oxygen reacts with the free radicals of resin materials, leading to premature termination of polymer chains, which interferes with hybrid layer formation and micromechanical interaction.^3^

The groups treated with argon plasma, regardless of oxygen association, exhibited inferior performance compared to the C group but superior performance compared to the LED and HP groups. Abreu et al.^27^ demonstrated that plasma treatment for 30 seconds can enhance adhesion by increasing hydrogen bonding interactions between collagen fibrils and the adhesive system. However, prolonged exposure may contribute to dentin degradation, potentially due to the reduction of phosphate species to carboxylic groups.^27^

These findings align with the present study, suggesting that the reduced bond strength values compared to the C group may be attributed to the 10-minute plasma exposure period.^28^ Additionally, the continuous and non-mechanized movement of specimens during plasma application complicates precise timing, considering variables such as beam diameter and total specimen area. This methodological limitation, combined with the lack of well-established parameters for this approach, may have influenced the results obtained.

The violet LED group also exhibited low bond strength values, a result supported by substrate analysis, which indicated the formation of a hydrophobic surface. The prolonged light exposure may have compromised the structural integrity of collagen fibrils, negatively affecting bond strength to this substrate. Barbosa et al.^19^ reported that even when violet LED is irradiated onto a thin enamel layer, there is a tendency for reduced collagen cohesive strength, similar to that observed in peroxide-treated teeth.

Additionally, except for the HP group, which exhibited extremely low bond strength at both aging periods, all treatments were affected by thermal cycling, resulting in a statistically significant reduction in adhesive strength. This deterioration can be attributed to differences in thermal expansion and hydrolytic degradation occurring during thermal cycling, impacting the presence of nanoleakage and degradation of the hybrid layer.^29^ This may explain the higher occurrence of adhesive failures and premature failures in all groups after the thermocycling. Consequently, the fifth null hypothesis was rejected, as thermal cycling influenced bond strength.

Although all the interventions reduced adhesive strength after thermal aging, the APL+O group showed a more pronounced drop (40%) in relation to the HP group (20%). This difference may be associated with prolonged exposure to oxygen plasma, which, despite improving initial wettability, can compromise dentin structure and the integrity of the hybrid layer. ^24^ ^,^ ^27^ ^,^ ^28^ ^,^ ^29^ ^,^ ^30^ The high generation of reactive oxygen species can deteriorate the organic matrix and collagen fibrils, which are essential for the integrity and stability of the adhesive interface.^12^ ^,^ ^19^ This damage, which may not be detected initially by the wettability results, tends to be manifested after thermocycling. A similar mechanism has already been reported in peroxide treatments, which reinforces the hypothesis of oxidative degradation in the performance of the APL+O group.

Bovine dentin was employed in this study because it is considered an accepted model for in vitro research, due to its availability, the possibility of standardizing the substrate, and suitable dimensions for specimen preparation. Although it has anatomical differences from human dentin, such as larger dentinal tubules, studies have demonstrated that its adhesive and mechanical behavior is comparable, which makes it a valid alternative for laboratory research.^31^ ^,^ ^32^

Thus, in the present study, while chromatic alteration produced promising results, both argon plasma (with or without oxygen) and violet LED compromised the effectiveness of the restoration system. Currently, no standardized protocol exists in the literature that simultaneously achieves dental bleaching while enhancing the adhesive strength of dental tissues. Furthermore, the variability in equipment and application of experimental protocols highlights the need for further research to develop peroxide-free techniques.

Future studies should explore argon plasma application at different exposure times and oxygen concentrations, its impact on collagen fibers and adhesive properties with various bonding systems, immediate color evaluation after application, and its effects on pulp health to develop clinically applicable protocols.

## Conclusion

This study concluded that argon plasma combined with oxygen exhibited whitening effectiveness comparable to conventional therapy and a greater ability to alter the dentin surface properties. The violet LED caused a significant temperature rise and made the surface hydrophobic. Furthermore, the whitening treatments directly applied to dentin tissue compromised the bond strength, and in general, thermal cycling compromised the bond strength of whitening treatments.
